# The Impact of the COVID-19 Pandemic on Diagnosis, Treatment, and Survival of Lung Cancer Patients in Thailand from 2019–2021

**DOI:** 10.3390/jcm15062277

**Published:** 2026-03-17

**Authors:** Chaiyut Charoentum, Chawin Aksorn, Khemruthai Chaiwipassatorn, Khwanchanok Siroros, Kittipich Tiangtawat, Thanika Ketpueak, Thatthan Suksombooncharoen, Busayamas Chewaskulyong

**Affiliations:** 1 Department of Internal Medicine, Oncology Unit, Faculty of Medicine, Chiang Mai University, Chiang Mai 50200, Thailand; thanika.k@cmu.ac.th (T.K.); thatthan.s@cmu.ac.th (T.S.); bchewask@gmail.com (B.C.); 2 Faculty of Medicine, Chiang Mai University, Chiang Mai 50200, Thailand; chawin_aksorn@cmu.ac.th (C.A.); fachaiwip@gmail.com (K.C.); bua.si@hotmail.com (K.S.); kuajaja@live.com (K.T.)

**Keywords:** COVID-19, lung cancer, waiting time, pandemic, MDT

## Abstract

**Background/Objectives**: The COVID-19 pandemic emerged in early 2020, disrupting global cancer services. We aimed to assess the pandemic’s impact on lung cancer diagnosis and treatment, including clinical characteristics, diagnostic methods, treatment patterns, and survival outcomes. **Methods**: A retrospective analysis of 1832 patients visiting Maharaj Nakorn Chiang Mai Hospital from January 2019 to December 2021 with suspected lung cancer was conducted. We evaluated demographic characteristics, diagnostic methods, treatment modalities, and survival results across this period. **Results**: Among the 698 eligible patients, the pandemic led to a 13% to 17% decline in newly diagnosed lung cancer cases. However, demographic and lung cancer characteristics, including age, gender, and smoking status, remained unchanged. The pandemic also saw an increase in asymptomatic cases and a 1.3 to 2.2 times higher occurrence of early-stage non-small cell lung cancer cases. The rapid implementation of healthcare policies prioritized the diagnosis of suspected cancer patients and maintained cancer care throughout the pandemic, resulting in similar diagnostic methods and waiting times compared to the pre-pandemic era. Treatment patterns displayed continuity, with a notable rise in 2 to 3 times higher surgical interventions and an 8% to 11% decrease in the initial delivery of palliative care. The delivery of systemic therapy for patients with advanced-stage disease was also maintained. One-year survival rates remained consistent across various lung cancer stages during the pandemic. **Conclusions**: The COVID-19 pandemic led to a modest decrease in new diagnoses, with limited effects on the demographic and clinical profiles of lung cancer cases. Survival rates among patients diagnosed during the pandemic remained stable compared to those diagnosed before the pandemic. These findings underscore the adaptability of the healthcare system, alongside the capabilities of multidisciplinary teams, in providing timely and effective diagnosis and ensuring uninterrupted essential treatment of lung cancer during challenging circumstances.

## 1. Introduction

The Coronavirus disease 2019 (COVID-19) pandemic significantly impacted global public health systems, leading to decreased capabilities in healthcare services, admissions, diagnoses, and therapeutics [1]. While healthcare systems inevitably increased demand for the care of COVID-19 patients and decreased availability for other diseases, maintaining prompt diagnosis and proper treatment services for other common, leading life-threatening conditions such as lung cancer is crucial [2]. Lung cancer remains the leading cause of cancer cases and deaths worldwide including in Thailand and has become a significant health problem in both genders due to its high morbidity and mortality rates [3,4]. It is well-recognized that an advanced-stage diagnosis, decline in performance status, and delayed treatment can greatly affect the survival of lung cancer patients [5,6]. The convergence of COVID-19 and lung cancer presents unique challenges for both patients and healthcare systems, necessitating a comprehensive understanding of the underlying interactions between these two entities.

Even under normal circumstances, the diagnostic pathways and management of lung cancer patients require complex coordination among healthcare providers and specialists, resulting in slow and non-resilient processes. The COVID-19 pandemic further exacerbated the situation by impacting access to regular healthcare services, leading to increased waiting times for hospital visits, diagnostic procedures, and lung cancer treatment. However, the full consequences of these delays are yet to be fully recognized.

There is limited research available on the specific impact of changes in assessment protocols, availability of diagnostic services, and shifts in health-seeking behavior during the COVID-19 pandemic on lung cancer. Early-stage diagnosis and timely treatment initiation are crucial for favorable outcomes. However, the pandemic caused potential delays in lung cancer diagnosis and management, which could adversely affect disease prognosis. Factors such as strained healthcare systems and patient hesitancy contribute to these delays. Further research is needed to understand and implement strategies to mitigate these effects, ensuring timely and effective care for lung cancer patients during crises, ultimately leading to improved outcomes.

## 2. Materials and Methods

This study conducted an observational retrospective cohort of newly diagnosed lung cancer patients at Maharaj Nakorn Chiang Mai Hospital, a facility affiliated with Chiang Mai University. Data collection spanned from 1 January 2019 to 31 December 2021. Ethical approval (Study code: MED-2564-08707) was obtained from the Research Ethics Committee, Faculty of Medicine, Chiang Mai University. Given the retrospective nature, patient consent was waived, but privacy and data protection were strictly maintained.

### 2.1. Study Objective

The primary objective of this study was to comprehensively assess the impact of the COVID-19 pandemic on various aspects of newly diagnosed lung cancer, including the number of new cases, patient characteristics, diagnostics, and treatment, during the pandemic years compared to the pre-pandemic period in 2019. The main aim of this study, however, was to investigate the COVID-19 pandemic’s effect on survival outcomes for those newly diagnosed with lung cancer, focusing on 2020 and 2021, and compared to the pre-pandemic period in 2019.

### 2.2. Sample Size

The sample size for this study was determined using the log-rank test, which is a survival analysis method that compares survivor functions between two groups. Previous studies conducted in Thailand reported an overall one-year survival rate ranging from 28% to 31% among patients diagnosed with lung cancer [7,8]. Additionally, another study revealed that a six-month delay in surgery led to a decrease in the five-year survival rate by 24.5% to 37.5% [9]. Based on these findings, we anticipated that the impact of the COVID-19 pandemic on diagnosis and treatment would result in an 8% to 10% reduction in the one-year survival rate. With a 95% confidence interval and a test power of 80%, the estimated sample size for each group ranged from 204 to 369 cases. To account for the potential loss to a follow-up rate of 10%, a total sample size of at least 612 cases was required to ensure the study’s statistical validity and meaningful conclusions.

### 2.3. Study Design

This study employed an observational retrospective cohort design using data from Maharaj Nakorn Chiang Mai Hospital’s electronic health database. The study population consisted of patients newly diagnosed with lung cancer, identified through the International Classification of Disease, Tenth Revision, and Clinical Modification codes (ICD-10) with code C34: Malignant neoplasm of bronchus and lung.

To establish a baseline, the reference group for this study was the “Pre-COVID-19 cohort” with patients diagnosed between 1 January 2019 and 31 December 2019. Thailand’s first COVID-19 case emerged on 13 January 2020, after which national public health measures were implemented, including the Emergency Decree on Public Administration issued on 26 March 2020. These measures included restrictions on foreign entry, high-risk area entry bans, limitations on travel and public gatherings, and social-distancing policies.

During the pandemic period, our tertiary referral hospital experienced intermittent increases in COVID-19 admissions, particularly during waves in 2020 and 2021. Some hospital resources were temporarily reallocated to support COVID-19 care, and operational adjustments were implemented, including reduced outpatient clinic capacity and rescheduling of selected elective procedures. However, diagnostic evaluation and treatment for suspected cancer cases were prioritized, and core oncology services remained operational throughout the study period.

The “COVID-19 cohort” included patients diagnosed from 1 January 2020, and 31 December 2020. Beginning in January 2021, the national COVID-19 vaccination program was progressively implemented in Thailand, initially targeting healthcare workers and high-risk groups, including cancer patients. Therefore, the COVID-19 with vaccination coverage cohort included patients diagnosed between 1 January 2021, and 31 December 2021.

Patients were included in this analysis if they met all of the following inclusion criteria: (1) newly diagnosed with lung cancer confirmed by pathological examination or radiographic diagnosis between 1 January 2019, and 31 December 2021, (2) aged 18 years or older, and (3) received their diagnosis and follow-up appointments at Maharaj Nakorn Chiang Mai Hospital.

We obtained data from the medical records, which included information such as the date of presentation, presenting symptoms, the interval between presentation and obtaining diagnostic procedures, and the interval between presentation and obtaining the final pathologic diagnosis and initiating the first lung cancer treatment. Patients who had recurrent lung cancer, metastatic cancer to the lung from other primary sites, or did not have a Thai national identification number to verify survival status and date of death by The Bureau of Registration Administration were excluded. General demographic data, presenting symptoms, clinical and tumor characteristics, diagnostic procedures, lung cancer treatment, and survival information were collected for all participants. Specifically, we recorded the date of the first hospital visit, the date of performing diagnostic procedures, the date of diagnosis confirmation, and the date of initiating lung cancer treatment to compare waiting times during each clinical process [10,11].

### 2.4. Statistical Analysis

The collected data were analyzed using commercial statistical software (STATA 17.0; Stata Corp LP, College Station, TX, USA). Descriptive statistics were performed to describe the characteristics of lung cancer patients in each cohort. Categorical descriptive statistics were presented as percentages while continuous descriptive statistics were presented as means with standard deviations (SDs) or medians with interquartile ranges, depending on the data distribution.

The primary analyses focused on survival outcome, while exploratory analyses were considered hypothesis-generating rather than confirmatory. Statistical analyses were conducted with two-tailed *p*-values < 0.05 considered statistically significant. Adjusted *p*-values were reported only for primary outcomes. Independent categorical analyses were performed using the Chi-square test or Fisher exact test. For the comparison of three independent continuous variables, one-way ANOVA (parametric) or the Kruskal–Wallis rank test (non-parametric) was utilized. Logistic regression, ordinal logistic regression, and multinomial logistic regression were employed for binary, ordinal, and categorical outcomes, respectively, to assess relationships between predictor and outcome variables. If the primary analyses yielded significant results, multiple comparison methods were applied as appropriate including Bonferroni’s Adjustment Method (parametric) to control the false discovery rate and Dunn’s test (non-parametric) for multiple group comparison. Poisson regression was performed to predict the cumulative survival rate at 1 year controlling for lung cancer stage and ECOG performance status.

In survival analysis, the survival probability among the three study groups (patients newly diagnosed with lung cancer in 2019, 2020, and 2021) was compared using the Log-rank test and presented with Kaplan–Meier plots, as well as median and restricted means of survival time (RMST). RMST was calculated using a 12-month time horizon, and adjusted comparisons between calendar-year cohorts were performed using an ANOVA-based regression approach, including stage at diagnosis and ECOG performance status as covariates, with survival times truncated at 12 months and censoring handled using standard RMST methods. Schoenfeld residuals were used to test for violation of the proportional hazard assumption.

Candidate variables were selected based on clinical relevance and significance in univariable analyses (*p* < 0.05). Missing data were handled using complete case analysis, whereby only patients with complete information for all variables included in the multivariable model were analyzed. Multivariable analysis was performed using the Cox proportional hazards regression model, and results were reported as adjusted hazard ratios (aHR). Backward stepwise selection was applied to derive the final multivariable model and identify independent predictors of mortality.

## 3. Results

Hospital service utilization during the study period. Overall hospital activity declined during the COVID-19 pandemic. The mean monthly number of outpatient visits decreased from 121,423 in 2019 to 112,868 in 2020 and 100,420 in 2021 (−7.0% and −17.3%, respectively; ANOVA *p* < 0.001), while inpatient admissions declined from 3486 to 3039 and 2922 (−12.8% and −16.2%; *p* < 0.05). In contrast, the total number of cancer treatments—including chemotherapy, radiation therapy, and palliative care—declined only modestly from 8691 in 2019 to 8512 in 2020 and 7989 in 2021 (−2.1% and −8.1%; *p* < 0.05). These findings suggest that oncology services were relatively maintained despite reductions in overall hospital utilization.

**Figure 1:**
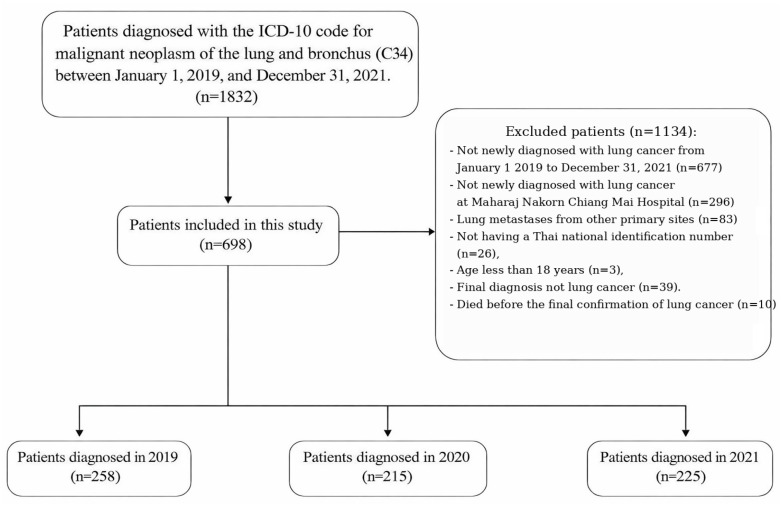
Patient flowchart.

A total of 1832 participants who visited Maharaj Nakorn Chiang Mai Hospital from 1 January 2019 to 31 December 2021 with suspected lung cancer (ICD-10 code C34) were identified. Among them, 698 patients met the inclusion criteria and were included in the final analysis. The number of excluded patients for various reasons is shown in Figure 1. The patients were categorized into three groups based on the year of lung cancer diagnosis. These groups consisted of 258 patients (36.96%) in the 2019 cohort, serving as the baseline before the COVID-19 pandemic, 215 patients (30.80%) in the 2020 cohort, and 225 patients (32.23%) in the 2021 cohort.

The general characteristics of the patients are presented in Table 1. The incidence of newly diagnosed lung cancer cases showed a significant reduction of 17% during the first year of the COVID-19 pandemic (2020), followed by a 13% decrease in the second year (2021). Despite this decline, no significant variations were found in the demographic profile of lung cancer patients, including their median age, gender distribution, smoking status, ECOG performance status, and the type of healthcare scheme affiliation, across the years 2019, 2020, and 2021.

The majority of patients, accounting for more than two-thirds, exhibited localized symptoms such as cough and shortness of breath, which remained consistent across the three-year study period (*p* = 0.858) However, there was a significant increase in the proportion of newly diagnosed lung cancer cases presenting with no symptoms during the two pandemic years, particularly in 2021 compared to both 2020 and 2019 (25.78%, 16.28%, 14.73% respectively; *p* = 0.005). The distribution of lung cancer subtypes status showed no significant variation over the study years. Notably, biomarker testing for lung cancer, specifically *EGFR* mutation analysis (using the single gene PCR method) and ALK detection (using immunohistochemical staining), was mainly reimbursed for patients with stage IV disease under the Civil Service Scheme during the study period. The proportion of lung cancer patients with positive common *EGFR* mutation (del 19 or L858R) and ALK-positive status remained relatively stable, ranging from 36% to 39% and 3.4% to 6.8%, respectively, over the three consecutive years. Of particular interest, there was a significant increase in the proportion of early-stage (stages I–II) non-small cell lung cancer (NSCLC) patients during the pandemic. In the first year of the COVID-19 pandemic, this proportion was 1.3 times higher, and in the year following the introduction of vaccinations, it was 2.2 times higher compared to the pre-pandemic year (*p* = 0.017). The proportion of patients diagnosed with small cell lung cancer remained small and stable, ranging from 6.2% to 7.9%, and were diagnosed at the limited stage in 17.6% to 28%, similar to the pre-pandemic year.

Table 2 displays the comparison of diagnostic methods for lung cancer in the years 2019, 2020, and 2021. The majority of cases were diagnosed using histopathologic evaluation, either alone or in conjunction with cytopathologic assessment, comprising 75.58% to 80.93% of the total cases. No significant differences were observed in the utilization of this diagnostic method across the three years (*p* = 0.372). Radiographic imaging alone was used as the diagnostic approach in only 8.37% to 12.40% of cases. Overall, the utilization of diagnostic methods remained consistent and did not significantly differ between the years.

The diagnostic procedures employed for lung cancer are detailed in Table 2. Fiberoptic bronchoscopy with trans-bronchial biopsy was the most frequently used diagnostic procedure across three consecutive years. The use of endobronchial ultrasound-guided biopsy (EBUS) showed a significant increase during the pandemic in 2020 (7.47%) and 2021 (11.49%) compared to 2019 (3.59%, *p* = 0.021). The remaining procedures for tissue diagnosis of lung cancer during the pandemic years were generally consistent with those observed in 2019. Thoracentesis was the most frequently used method for obtaining cytopathologic specimens, and its utilization remained stable throughout the three years. Notably, pericardiocentesis was only performed in 2020, with no cases in 2019 and 2021. Overall, the utilization of diagnostic methods remained consistent and did not significantly differ between the years.

Table 3 presents the delays observed in the clinical process for the diagnosis and treatment of lung cancer. The table demonstrates various time intervals and their comparisons across 3 years. The median time interval from the onset of symptoms to the first hospital visit remained consistent at approximately 31 days in all three years, with no statistically significant difference observed. Similarly, the median time intervals from the first hospital visit to performing a tissue biopsy, obtaining a cytological sample, and obtaining imaging studies showed no significant variation across the three years. Notably, the median time interval for obtaining a histopathologic diagnosis following a tissue biopsy exhibited a significant reduction in 2021 compared to the pre-COVID-19 baseline of 2019 (7 days vs. 15 days, *p* = 0.001). Conversely, the median time interval between the first hospital visit and the diagnosis displayed a statistically significant increase in 2020 compared to 2019 (30 days vs. 27 days, *p* = 0.039), indicating a potential disruption in the diagnostic process during the initial year of the COVID-19 pandemic. However, in 2021, the median time interval then decreased slightly compared to 2019. Regarding the median waiting time from diagnosis to the initiation of the first treatment, no substantial variations were observed across the three consecutive years, ranging from 17.5 days to 23 days. The median time intervals between diagnosis and the receipt of different treatment modalities showed no significant changes, except for chemotherapy, which exhibited a marginally significant increase in the median time interval in 2021 compared to 2019 (*p* = 0.049).

The most common initial treatment administered to patients was standard systemic cytotoxic chemotherapy, followed by targeted therapy with *EGFR* TKI. Immunotherapy, either alone or in combination with chemotherapy, was exclusively given to patients participating in clinical trials, and there was a higher participation rate in clinical trials during the pandemic years, as shown in Table 3. Interestingly, there was a significant increase in the use of surgical treatment during the COVID-19 pandemic, with a twofold increase in 2020 and a threefold increase in 2021 compared to 2019 (5.81% vs. 10.23% vs. 14.67%; *p* = 0.005). Conversely, the delivery of only palliative treatment as the initial treatment significantly decreased during the pandemic years, with rates of 29.7%, 21.4%, and 18.67% in 2019, 2020, and 2021, respectively (*p* = 0.019). The administration rates of other initial treatments remained consistent throughout the pandemic period (Table 3).

**Figure 2:**
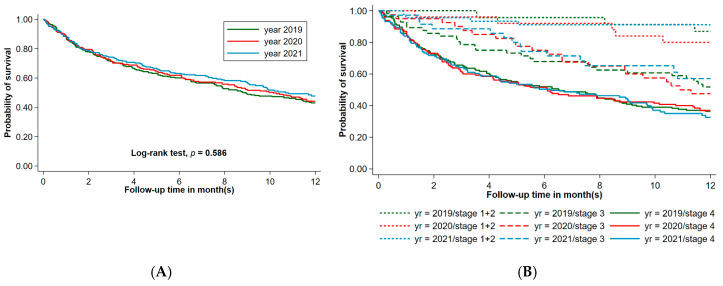
(**A**) Illustrated Kaplan–Meier Analysis of 1-year overall survival of lung cancer patients in 2019, 2020, and 2021. (**B**) Illustrated Kaplan–Meier Analysis of 1-year overall survival of lung cancer patients by stage in 2019, 2020, and 2021.

We observed similar one-year survival rates among lung cancer patients in 2019, 2020, and 2022, with rates of 43.02%, 44.19%, and 51.11%, respectively (*p* = 0.168), as shown in Table 4. The median survival times for lung cancer patients in 2019, 2020, and 2021 were 8.80, 10.15, and 10.68 months, respectively, with no significant differences observed (*p* = 0.586), as depicted in Figure 2A. The restricted mean survival times (RMST) were 7.50 months (95% CI, 6.93–8.07), 7.67 months (95% CI, 7.04–8.29), and 7.92 months (95% CI, 7.30–8.53), respectively, with no statistically significant difference across the three consecutive years. After adjusting for stage, performance status, and calendar year, the adjusted RMST difference showed similar results, with no statistically significant differences observed. These findings suggest that after controlling for key prognostic factors, no significant difference in RMST was observed across the three years. The one-year survival rates of lung cancer patients, stratified by stage alone or adjusted by both stage and performance status, followed a similar trend across the three consecutive years, with no statistically significant differences observed (Figure 2B). The one-year cumulative survival rates for patients by stage alone or adjusted by both stage and performance status with stages I–II, III, and IV in 2019, 2020, and 2021 ranged from 80% to 91%, 47.5% to 60%, and 35.17% to 37.77%, respectively. Exploratory analyses stratified by month of diagnosis did not reveal consistent survival differences across calendar years (Supplementary Table S1 and Figure S1).

We analyzed the baseline clinical factors and their correlation with patient mortality, as shown in Table 5. Our study revealed several significant associations between baseline clinical factors and higher mortality rates. Specifically, advanced age (75 years or older), a history of smoking, male gender, the presence of symptoms at presentation, squamous carcinoma histology, an ECOG performance status of 2 or higher, negative *EGFR* mutation status, and locally advanced or advanced stages were all identified as significant factors associated with increased mortality in the univariate analysis. Furthermore, our subsequent multivariable Cox analysis confirmed that male gender, the presence of local symptoms or symptoms from distant metastasis at presentation, squamous histology, an ECOG performance status of 2 or higher, stage IV disease, and receiving only palliative care without cancer-specific treatment were strong independent predictors of higher mortality. An interesting finding was the apparent association between a longer diagnosis-to-treatment interval (≥28 days) and lower mortality. This observation should be interpreted cautiously, as it likely reflects reverse causation related to clinical triage rather than a protective effect of treatment delay. In stratified analyses by stage, this association was primarily observed in advanced-stage disease but not in early-stage disease (stages I–II), suggesting that the finding may be driven by confounding from disease severity and treatment prioritization rather than a causal effect of treatment delay.

## 4. Discussion

The present study aimed to investigate various aspects of lung cancer diagnosis and treatment in the context of the COVID-19 pandemic. Our findings provide valuable insights into the impact of the pandemic on lung cancer management, encompassing clinical characteristics, diagnostic methods, treatment patterns, and survival outcomes.

During the pandemic, we observed a reduction in the number of newly diagnosed lung cancer cases, with a decrease ranging from 13% to 17%. This decline is likely due to various factors related to how patients perceive their health and the functioning of healthcare services during this period [2,12]. Similar decreases in lung cancer diagnoses during the pandemic have been reported in other countries such as Canada and Italy during the first year of the pandemic, corroborating our findings [13,14]. Patient-related factors such as fear of COVID-19 transmission and self-isolation during the outbreak may have led to delayed healthcare-seeking behavior and a decrease in the number of patients seeking medical attention for symptoms suggestive of lung cancer [15]. Additionally, system-related factors such as emergency decrees and healthcare restructuring might have restricted access to medical facilities and diverted healthcare resources to COVID-19 management [16]. These combined factors likely contributed to the overall decrease in newly diagnosed lung cancer cases observed during the pandemic. However, it is important to note that studies on new lung cancer diagnoses during the pandemic reported varying degrees of decline in incidence compared to the pre-pandemic period. These variations can be attributed to differences in local healthcare system policies, public health measures, and patient populations.

Despite this decrease in incidence, the demographic profile of lung cancer patients and the characteristics of our lung cancer cases remained largely unchanged. This suggests that the pandemic and subsequent vaccination efforts had limited influence on the overall composition of lung cancer patients in terms of age, gender, symptom presentation, histologic sub-type distribution, and *EGFR* mutation status. Notably, we did observe an increase in the proportion of asymptomatic cases and a 1.3- to 2.2-fold higher occurrence of early-stage non-small cell lung cancer (NSCLC) cases during the pandemic. The exact cause of this finding remains unclear. One possible explanation is that institutional and national healthcare policies in our setting prioritized diagnostic evaluation for suspected cancer cases, allowing lung cancer diagnostic services to remain accessible. In addition, increased use of chest imaging during the pandemic, including imaging performed for respiratory symptoms or evaluation of suspected COVID-19 infection, may have contributed to the incidental detection of asymptomatic lung nodules and early-stage lung cancers. This observation may also represent an “incidental detection effect”, whereby increased chest imaging during the pandemic led to the identification of otherwise asymptomatic lung nodules and early-stage lung cancers [17,18]. In contrast, studies in Ireland, Italy, and Spain observed that lung cancer patients were more likely to be diagnosed in a more advanced stage and worsening performance status at presentation [19,20,21,22]. However, this finding likely reflects local healthcare system adaptations at our tertiary referral center and should therefore be interpreted cautiously, as it may not be generalizable to other healthcare settings.

During the COVID-19 pandemic, lung cancer management in Thailand was adapted to balance infection control measures with the need to maintain essential oncology services. Institutional practice emphasized prioritizing patients with potentially curable disease for timely diagnostic staging and curative-intent treatment, while systemic therapy and radiotherapy services for advanced disease were largely maintained. In terms of diagnostic approaches, we found no significant variation compared to the pre-pandemic period. Following the implementation of social-distancing measures in early 2020, our institution quickly adapted by implementing telemedicine and teleconferencing to support several clinical pathways in lung cancer care. Our weekly face-to-face multidisciplinary thoracic tumor (MDT) conference and consultations were shifted to an online platform, resulting in the highest level of participants recorded [23]. Despite the COVID-19 pandemic, the waiting time from symptoms to diagnosis in our study remained less than one month, and the interval from sample collection to diagnosis was less than 14 days, aligning with the recommended wait times reported in the literature during non-pandemic periods [10,11].

We did observe a significant delay in the interval between the first hospital visit and diagnosis during the initial year of the pandemic in 2020 compared to 2019. However, once the Ministry of Public Health of Thailand imported and prioritized the limited supply of COVID-19 vaccines for healthcare personnel, hospital services fully resumed, and we no longer observed this delay in the subsequent year of 2021. Overall, our diagnostic infrastructure demonstrated resilience and adaptability by implementing multidisciplinary adjustments and utilizing online platforms for telemedicine and teleconferences. While there were some delays in waiting times during the initial year, the overall diagnostic process was efficiently maintained and even expedited in the subsequent year. As a result of closer online consultations and multidisciplinary conferences with increased participation of pulmonologists in recent years, we also observed a significantly higher prevalence of endobronchial ultrasound (EBUS)-guided needle aspiration. Because EBUS is frequently used for mediastinal staging and nodal evaluation in patients with potentially resectable lung cancer and has largely replaced mediastinoscopy in our institution, the increased use of EBUS may reflect the greater number of patients diagnosed at early stages and evaluated for curative-intent surgical treatment.

Regarding treatment patterns, the majority of lung cancer-specific therapies were preserved during the pandemic. Notably, there was a significant increase in the utilization of surgical treatment for lung cancer patients, indicating a shift in treatment priorities during the pandemic. Our local and national policies prioritized the provision of services and treatment for patients with suspected new cancer diagnoses or those requiring ongoing cancer treatment, ensuring uninterrupted and timely care throughout the crisis. Additionally, there was a notable decrease in the delivery of palliative treatment as the initial approach, highlighting a greater emphasis on active and potentially curative treatment strategies during the pandemic. Patients with advanced-stage lung cancer who required treatment, including systemic therapy with chemotherapy, targeted therapy, or participation in first-line immunotherapy clinical trials, were able to receive these treatments without significant delays throughout the pandemic.

These adaptations may explain why, despite reductions in the overall hospital utilization, the cancer treatment volume declined only modestly and short-term survival outcomes remained relatively stable during the study period. Similar findings have been reported in a public tertiary hospital in Spain, where healthcare system resilience and streamlined care pathways proved critical for sustaining cancer outcomes during the COVID-19 crisis, consistent with our observations [24].

Importantly, our study revealed that the one-year survival rates and RMST for lung cancer patients remained relatively stable across different stages during the COVID-19 pandemic. Adjusted RMST analyses incorporating stage at diagnosis, performance status, and calendar year yielded consistent findings, indicating no statistically significant survival differences after controlling for key prognostic factors. Month-specific analyses were conducted as exploratory assessments to evaluate potential seasonal variation; given the large number of comparisons and small monthly sample sizes, these results should be interpreted cautiously. We acknowledge that much of the existing literature assessing the impact of the COVID-19 pandemic on cancer care has relied on monthly or pre-/post-lockdown analyses, which are well-suited to detecting abrupt changes in healthcare utilization following public health restrictions. Such studies have reported heterogeneous patterns across cancer sites, including immediate declines with rapid recovery, delayed recovery, or minimal impact. In contrast, our study focused on annual cohorts to capture the cumulative and sustained effects of the pandemic on lung cancer care within a tertiary referral center, reflecting adaptive responses in diagnostic pathways, treatment prioritization, and multidisciplinary coordination that evolved throughout 2020 and 2021. We recognize that this approach may attenuate short-term fluctuations associated with specific lockdown periods and may therefore underestimate acute disruptions observed at the monthly level. However, it provides complementary insight into the net effect of the pandemic on lung cancer outcomes after health systems had adapted to ongoing constraints. Overall, our findings suggest that the COVID-19 pandemic did not have a significant adverse effect on short-term survival outcomes in lung cancer patients treated at our institution, highlighting the adaptability of local lung cancer care pathways in maintaining access to essential diagnosis and treatment during a period of substantial healthcare disruption.

We observed several factors that were significantly associated with poor survival outcomes, including the presence of symptoms at presentation, whether local or from distant metastasis, poor ECOG performance status, advanced-stage diseases, and receiving only palliative care. These findings align with previous studies in the literature [25]. There have been conflicting results regarding the association between waiting time and prognosis in patients diagnosed with different stages of lung cancer in the pre-pandemic literature [10]. In our study, we found that longer waiting times from diagnosis to treatment, specifically exceeding 28 days, were associated with significantly lower mortality in patients predominantly with advanced-stage disease. This is consistent with several prior studies [26,27]. These findings may suggest that patients with more symptomatic and advanced-stage diseases received treatment sooner after diagnosis, as the treatment decision and testing for these patients may be more straightforward for the multidisciplinary team, potentially resulting in shorter waiting times. However, due to the limited number of patients with early-stage disease in our study, we were unable to further test this hypothesis.

Several limitations should be considered when interpreting the findings of this study. First, the retrospective design led to some missing data, which may have affected the comprehensiveness of our analysis. Second, as an observational retrospective cohort study conducted in a real-world setting, our findings demonstrate associations rather than causal relationships. Furthermore, the relatively small sample size limited our ability to conduct simultaneous comparisons across multiple pandemic years, restricting the analysis primarily to pairwise comparisons between each pandemic year and the pre-pandemic baseline. A larger, multi-institutional cohort would be required for more robust trend analyses. In addition, because analyses were conducted at the annual level, the study was not designed to detect abrupt short-term changes in cancer diagnosis or treatment initiation immediately following lockdown implementation, which may be better captured using monthly time-series or interrupted time-series designs.

Our analysis assumed a relatively stable increasing trend in lung cancer incidence in recent years and used 2019 as the pre-pandemic baseline due to incomplete data from earlier years. Although this approach allowed for the evaluation of healthcare system adaptations during the pandemic, it may have attenuated short-term disruptions that occurred early in 2020. The modest sample size, multiple comparisons, and single-center design may also limit the generalizability of our findings to other healthcare systems with different pandemic responses. Therefore, exploratory analyses should be interpreted cautiously as hypothesis-generating. Although Bonferroni correction was applied to control for multiple testing, larger sample sizes or alternative statistical approaches could further strengthen the validity of the findings. Another limitation is that survival outcomes were limited to 1-year overall survival, which may not fully reflect the long-term prognosis of patients with early-stage NSCLC, where 3–5-year survival is more clinically meaningful. Longer follow-up will therefore be required to determine whether delayed effects of the COVID-19 pandemic on diagnosis, treatment patterns, or survival emerge over time.

Finally, our study included only lung cancer patients evaluated by the multidisciplinary team at Maharaj Nakorn Chiang Mai Hospital; thus, the results may be most applicable to similar healthcare settings [23,24]. The true number of new lung cancer cases during the pandemic may also have been underestimated, particularly among individuals who never presented to our healthcare system. Additionally, as the COVID-19 pandemic was evolving, limited evidence on optimal local management early in the pandemic may have influenced treatment decisions and outcomes.

## 5. Conclusions

In conclusion, the COVID-19 pandemic had a limited impact on the demographic profile and clinical characteristics of lung cancer patients in our institution. Although the number of newly diagnosed cases declined, diagnostic and treatment services were largely maintained through institutional adaptations, including telemedicine-supported multidisciplinary care. Treatment strategies remained largely consistent, with increased surgical management and the sustained delivery of active therapies, while short-term survival outcomes remained stable. As this study reflects the early pandemic period (2019–2021), the findings provide important insight into healthcare system adaptations and the resilience of oncology services during a major public health crisis. Further studies with longer follow-up and more recent data are needed to evaluate the long-term impact of the pandemic on lung cancer diagnosis, treatment patterns, and survival outcomes.

## Figures and Tables

**Table 1:** Characteristics of lung cancer diagnoses.

Characteristics	2019	2020	2021	*p*-Value
Number of patients	258	215	225	
Age (Mean ± SD)	66.21 ± 10.50	65.16 ± 10.11	67.38 ± 10.62	0.082
Gender				0.639
Male	148 (57.36)	114 (53.02)	125 (55.56)	
Female	110 (42.64)	101 (46.98)	100 (44.44)	
Smoking status				0.184
Smoker	33 (14.54)	40 (20.62)	35 (18.23)	
Non-smoker	75 (33.04)	74 (38.14)	72 (37.50)	
Ex-smoker	119 (52.42)	80 (41.24)	85 (44.27)	
Missing data	31	21	33	
ECOG				0.820
0–1	141 (57.32)	123 (59.71)	123 (58.02)	
2	77 (31.30)	61 (29.61)	59 (27.83)	
More than 2	28 (11.38)	22 (10.68)	30 (14.15)	
Missing data	12	9	13	
Thailand’s health financing scheme				0.373
Civil Service Scheme	136 (52.71)	117 (54.42)	137 (60.89)	
Social Security Scheme	13 (5.04)	14 (6.51)	9 (4.00)	
Universal Coverage Scheme	99 (38.37)	79 (36.74)	76 (33.78)	
Private funding	10 (3.88)	5 (2.33)	3 (1.33)	
Symptoms at presentation				0.0045
Asymptomatic	38 (14.73)	35 (16.28)	58 (25.78)	
Symptomatic	220 (85.27)	180 (83.72)	167 (74.22) **,†	
Type of symptoms				0.858
Local symptoms	158 (71.82)	124 (68.69)	118 (70.66)	
Loco-regional symptoms	10 (4.55)	4 (2.22)	5 (2.99)	
Distant metastatic	52 (23.64)	51 (28.33)	43 (25.75)	
Paraneoplastic syndrome	0	1 (0.56)	1 (0.60)	
Sub-type of lung cancer NSCLC				0.4248
Non-Squamous cell CA	196 (75.97)	158 (73.49)	178 (79.11)	
Squamous cell CA	37 (14.34)	40 (18.60)	33 (14.67)	
Small cell CA	25 (9.69)	17 (7.91)	14 (6.22)	
Stage of NSCLC				0.017
Stage I	13 (5.58)	20 (10.26)	29 (13.74) *	
Stage II	10 (4.29)	5 (2.56)	16 (7.58)	
Stage III	56 (24.03)	40 (20.51)	35 (16.59)	
Stage IV	154 (66.09)	130 (66.67)	131 (62.09)	
Stage of SCLC				0.744
Limited disease	5 (20.00)	3 (17.65)	4 (28.57)	
Extensive disease	20 (80.00)	14 (82.35)	10 (71.43)	
*EGFR* mutation status				0.797
Negative	107 (64.46)	90 (60.81)	96 (63.16)	
Positive	59 (35.54)	58 (39.19)	56 (36.84)	
Unknown	92	67	73	
ALK				0.426
Negative	155 (93.37)	143 (96.62)	143 (94.08)	
Positive	11 (6.63)	5 (3.38)	9 (5.92)	
Unknown	92	67	73	

Pairwise comparisons were performed using Bonferroni’s method. Comparison with 2019: * *p* < 0.05, ** *p* < 0.01. Comparison with 2020: † *p* < 0.05.

**Table 2:** Diagnostic procedures for lung cancer.

**Diagnostic Methods**		**2019** **(*n* = 258)**		**2020** **(*n* = 215)**		**2021** **(*n* = 225)**	***p*-Value**
		**(*n*, %)**		**(*n*, %)**		**(*n*, %)**	
By histopathologic with or without cytopathologic proven		195 (75.58)		174 (80.93)		174 (77.33)	0.372
By cytopathologic proven		31 (12.02)		23 (10.70)		30 (13.33)	0.698
By radiographic imaging		32 (12.40)		18 (8.37)		21 (9.33)	0.314
**Procedure**	***n***	**2019** **(*n* = 195)**	***n***	**2020** **(*n* = 174)**	***n***	**2021** **(*n* = 174)**	***p*** **-value**
Bronchoscopy with endobronchial biopsy or transbronchial needle aspiration	79	79 (40.51)	65	65 (37.36)	66	66 (37.93)	0.800
Endobronchial ultrasound (EBUS) guided needle aspiration	7	7 (3.59)	13	13 (7.47)	20	20 (11.49) *	0.021
Transthoracic needle aspiration	23	23 (11.79)	23	23 (13.22)	27	27 (15.52)	0.577
Video-assisted thoracoscopic surgery (VATS)	43	43 (22.05)	28	28 (16.09)	31	31 (17.82)	0.319
Mediastinoscopy	1	1 (0.51)	2	2 (1.15)	1	1 (0.57)	0.751
Open surgery	6	6 (3.08)	6	6 (3.45)	3	3 (1.72)	0.595
Thoracotomy		4 (66.67)		2 (33.33)		1 (33.33)	
Craniotomy		2 (33.33)		4 (66.67)		2 (66.67)	
Pleural biopsy	11	11 (5.64)	6	6 (3.45)	1	1 (0.57) *	0.070
Lymph node biopsy	19	19 (9.74)	22	22 (12.64)	14	14 (8.05)	0.360
Others tissue biopsy	11	11 (5.64)	13	13 (7.47)	13	13 (7.47)	0.721
**Procedures to obtain cytopathologic specimens for diagnosis of lung cancer**							
**Procedure**	***n***	**2019** **(*n* = 31)**	***n***	**2020** **(*n* = 23)**	***n***	**2021** **(*n* = 30)**	***p*** **-value**
Thoracentesis	22	22 (70.97)	12	12 (52.17)	18	18 (60.00)	0.364
Lumbar puncture	0	0	0	0	1	1 (3.33)	0.732
Bronchoscopy with bronchial washing	6	6 (19.35)	1	1 (4.35)	5	5 (16.67)	0.328
Bronchoscopy with bronchial brushing	1	1 (3.23)	1	1 (4.35)	2	2 (6.67)	0.820
Pericardiocentesis	0	0	3	3 (13.04)	0	0	0.127
Percutaneous fine needle aspiration (FNA)	3	3 (9.68)	6	6 (26.09)	4	4 (13.33)	0.256

For small cell count Firth’s logistic regression was used. Pairwise comparisons were performed using Bonferroni’s method. Comparison with 2019: * *p* < 0.05.

**Table 3:** Healthcare waiting time for lung cancer patients.

Interval (Days)	2019		2020		2021		*p*-Value
	*n*	Median (IQR) Days	*n*	Median (IQR) Days	*n*	Median (IQR) Days	
Symptoms onset to first hospital visit	220	31 (13.50, 61)	180	31 (14, 62)	167	31 (15, 61)	0.550
First hospital visit to perform diagnostic procedures	258		215		225		
Tissue biopsy	195	16 (6, 37)	174	20.5 (7, 38)	174	20 (6, 43)	0.383
Cytological sample imaging	31	5 (0, 18)	23	11 (1, 17)	30	7.5 (3, 29)	0.420
CT scan	31	8 (5, 14)	18	24.5 (8, 39) *	21	12 (4, 30)	0.066
MRI	1	1 (1, 1)	0	-	0	-	-
Performing diagnostic procedures for histopathologic diagnosis							
Tissue biopsy	195	15 (8, 23)	174	13 (7, 19)	174	7 (5, 8) **,††	0.001
Cytological sample	31	8 (4, 18)	23	7 (5, 14)	30	7.5 (4, 10)	0.524
First hospital visit to diagnosis	258	27 (15, 46)	215	30 (19, 51)	225	25 (11, 47) †	0.039
Diagnosis to first treatment	241	23.0 (9, 42)	196	17.5 (4.5, 36)	213	23 (8, 45)	0.151
Diagnosis to first treatment							
Surgery	15	16 (4, 39)	22	27 (13, 47)	33	32 (20, 53)	0.171
Chemotherapy	94	22.5 (10, 48)	86	15 (8, 35)	86	27.5 (14, 45) †	0.049
Immunotherapy + chemo.	6	60.5 (38, 84)	3	34 (28, 69)	12	55.5 (31, 77)	0.506
Targeted therapy	40	31.5 (18, 46.5)	32	35.5 (20.5, 49)	30	24 (17, 38)	0.158
Radiotherapy	11	24 (16, 70)	7	17 (15, 24)	10	17 (12, 48)	0.278
Palliative treatment	75	15 (7, 32)	46	16 (2, 28)	42	12.5 (4, 29)	0.446
Missing	17	-	19	-	12	-	-

Comparison with 2019: * *p* < 0.05, ** *p* < 0.001. Comparison between 2020 and 2021: † *p* < 0.05, †† *p* < 0.001.

**Table 4:** Survival outcome of lung cancer patients in 2019, 2020 and 2021.

Survival	2019 (*n* = 258)	2020 (*n* = 215)	2021 (*n* = 225)	*p*-Value
Median survival time (months)	8.80	10.15	10.68	0.586 *
Restricted mean survival time (months)				
Unadjusted restricted mean survival time	7.50 (6.93, 8.07)	7.67 (7.04, 8.29)	7.92 (7.30, 8.53)	
Difference from year 2019 (months, 95% CI, *p*-value)		0.164 (−0.685, 1.013) *p* = 0.705	0.415 (−0.425, 1.255) *p* = 0.333	-
Difference from year 2020 (months, 95% CI, *p*-value)			0.251 (−0.627, 1.129) *p* = 0.576	
Adjusted RMST for stage of NSCLC and performance status (95% CI)				
Difference from year 2019		−0.142 (−0.938, 0.653) *p* = 0.726	0.310 (−0.462, 1.082) *p* = 0.431	
Difference from year 2020			0.453 (−0.368, 1.273) *p* = 0.279	
1-year cumulative survival rate, *n* (%)	111 (43.02)	95 (44.19)	115 (51.11)	0.168
1-year cumulative survival rate by stage, *n* (%)	2019 (*n* = 233)	2020 (*n* = 195)	2021 (*n* = 211)	
Stages I–II	20 (86.96%)	20 (80.00%)	41 (91.11%)	0.561
Stage III	29 (51.79%)	19 (47.50%)	21 (60.00%)	0.833
Stage IV	56 (36.36%)	48 (36.92%)	47 (35.88%)	0.899
1-year cumulative survival rate adjusted for stage of NSCLC and performance status (95% CI)				
Stages I–II	72.10 (60.92, 83.27)	67.01 (55.68, 78.34)	78.58 (69.99, 87.17)	0.224
Stage III	47.50 (38.45, 56.54)	44.14 (34.59, 53.70)	51.77 (41.69, 61.85)	0.244
Stage IV	39.81 (33.59, 46.04)	37.00 (30.53, 43.47)	43.39 (36.77, 50.02)	0.242

* Log rank test.

**Table 5:** Subgroup analysis of mortality of patients with lung cancer.

Characteristics	Total	Died (%)	Univariate Analysis			Cox Regression		
			HR	95% CI	*p*-Value	AHR	95% CI	*p*-Value
Age (years)								
<60	167	84 (50.30)	Ref.					
60–74	390	202 (51.79)	1.10	0.85–1.42	0.456			
≥75	141	91 (64.54)	1.62	1.20–2.18	0.001			
Smoking status								
Non-smoker	221	91 (41.18)	Ref.			Ref.		
Smoker	108	77 (71.30)	2.44	1.80–3.30	0.000	1.47	0.99–2.19	0.057
Ex-smoker	284	164 (57.75)	1.61	1.25–2.08	0.000	0.94	0.67–1.31	0.715
Sex								
Female	311	139 (44.69)	Ref.			Ref.		
Male	387	238 (61.50)	1.59	1.29–1.96	0.000	1.49	1.08–2.06	0.016
Symptom onset at presentation								
No symptoms	131	29 (22.14)	Ref.			Ref.		
Local symptoms	400	234 (58.50)	3.37	2.29–4.96	0.000	3.29	1.02–10.60	0.046
Locoregional symptoms	19	9 (47.37)	2.43	1.15–5.13	0.020	2.28	0.55–9.40	0.253
Distant metastatic symptoms	146	104 (71.23)	5.13	3.39–7.74	0.000	3.42	1.03–11.36	0.045
Paraneoplastic syndrome	2	1 (50.00)	2.46	0.33–18.02	0.377			
Stage of NSCLC								
I–II	93	12 (12.90)	Ref.			Ref.		
III	131	62 (47.33)	4.29	2.31–7.96	0.000	2.19	0.77–6.25	0.142
IV	415	264 (63.61)	7.36	4.13–13.14	0.000	3.27	1.15–9.33	0.027
Type of lung cancer								
Non-Squamous CA	532	259 (48.68)	Ref.			Ref.		
Squamous cell CA	110	79 (71.82)	1.78	1.38–2.29	0.000	1.82	1.28–2.58	0.001
Small cell CA	56	39 (69.64)	1.69	1.21–2.37	0.002	-	-	-
*EGFR* mutation status								
Negative	293	159 (54.27)	Ref.					
Positive	173	60 (34.68)	0.53	0.39–0.71	0.000			
ECOG								
0–1	387	150 (38.76)	Ref.			Ref.		
2	197	133 (67.51)	2.59	2.05–3.28	0.000	1.80	1.31–2.47	0.000
3–4	80	72 (90.00)	5.60	4.20–7.46	0.000	2.45	1.58–3.78	0.000
First treatment type								
Surgery	70	7 (10.00)	0.03	0.01–0.06	0.000	0.18	0.06–0.53	0.002
Standard chemotherapy	266	127 (47.74)	0.17	0.13–0.22	0.000	0.22	0.16–0.32	0.000
Immunotherapy + chemotherapy	21	6 (28.57)	0.10	0.04–0.22	0.000	0.17	0.07–0.41	0.000
Targeted therapy	102	33 (32.35)	0.11	0.07–0.16	0.000	0.14	0.08–0.23	0.000
Palliative treatment	163	152 (93.25)	Ref.			Ref.		
Radiotherapy	28	15 (53.57)	0.24	0.14–0.41	0.000	0.24	0.12–0.48	0.000
Interval from symptom to diagnosis								
<28 days	74	42 (56.76)	Ref.			Ref.		
28–83 days	298	193 (64.77)	1.27	0.91–1.77	0.160	1.06	0.69–1.65	0.786
≥84 days	213	116 (54.46)	0.93	0.65–1.33	0.695	0.79	0.49–1.28	0.340
Interval from diagnosis to first treatment								
<28 days	367	226 (61.58)	Ref.			Ref.		
≥28 days	284	115 (40.49)	0.50	0.40–0.63	0.000	0.57	0.43–0.75	0.000

## Data Availability

The data that support the findings of this study are available from the corresponding author, C.C., ccharoentum@gmail.com, upon reasonable request. The data are not publicly available due to privacy or ethical restrictions.

## Supplementary Materials

The following supporting information can be downloaded at: https://www.mdpi.com/article/10.3390/jcm15062277/s1, Figure S1: Monthly variation in 1-year survival rates by year of diagnosis (2019–2021); Table S1: Monthly variation in 1-year survival and year-to-year comparisons by diagnosis year (2019–2021).

## Abbreviations

The following abbreviations are used in this manuscript:

aHR	Adjusted Hazard Ratios
COVID-19	The Coronavirus disease 2019
EBUS	Endobronchial Ultrasound-Guided Biopsy
ICD-10	International Classification of Diseases and Related Health Problem 10th Revision
LSD	Least Significant Difference
NSCLC	Non-Small Cell Lung Cancer
MDT	Multidisciplinary Thoracic Tumor
SD	Standard Deviation
