# Supplementary material for: The Impact of the COVID-19 Pandemic on Diagnosis, Treatment, and Survival of Lung Cancer Patients in Thailand from 2019–2021
Source: J Clin Med. 2026 Mar 17;15(6):2277. doi: 10.3390/jcm15062277 (PMC13026722; doi:10.3390/jcm15062277)
Supplement: Supplementary file 1 [file jcm-15-02277-s001.zip › jcm-4180576-supplementary.pdf]

Table Regends

Supplementary Table S1. Monthly variation in 1-year survival and year-to-year comparisons by diagnosis year (2019–2021).

Figure S1. Monthly Variation in 1-Year Survival Rates by Year of Diagnosis (2019–2021)

Supplementary Table S1. Monthly variation in 1-year survival and year-to-year comparisons by diagnosis year (2019–2021).

	Year of Diagnosis									Compare 1-year Survival rate					
	2019			2020			2021			2020 vs 2019		2021 vs 2019		2021 vs 2020	
Month of diag.	Total	Alive	1 year Surv. rate	Total	Alive	1 year Surv. rate	Total	Alive	1 year Surv. rate	OR* (95% CI)	p value	OR* (95% CI)	p value	OR* (95% CI)	p value
1	16	2	12.50	6	2	33.33	21	8	38.10	3.50 (0.37, 33.31)	0.276	4.31 (0.77, 24.14)	0.097	1.23 (0.18, 8.33)	0.831
2	17	6	35.29	18	8	44.44	13	4	30.77	1.47 (0.38, 5.72)	0.581	0.81 (0.17, 3.81)	0.795	0.56 (0.12, 2.49)	0.443
3	21	11	52.38	28	11	39.29	28	15	53.57	0.59 (0.19, 1.85)	0.363	1.05 (0.34, 3.26)	0.934	1.78 (0.62, 5.15)	0.286
4	24	14	58.33	20	12	60.00	21	11	52.38	1.07 (0.32, 3.59)	0.911	0.79 (0.24, 2.56)	0.689	0.73 (0.21, 2.53)	0.624
5	17	7	41.18	15	8	53.33	11	6	54.55	1.63 (0.40, 6.63)	0.493	1.71 (0.37, 7.92)	0.490	1.05 (0.22, 5.00)	0.951
6	24	7	29.17	20	6	30.00	20	13	65.00	1.04 (0.28, 3.82)	0.952	4.51 (1.26, 16.10)	0.020	4.33 (1.15, 16.32)	0.030
7	24	11	45.83	16	7	43.75	28	16	57.14	0.92 (0.26, 3.28)	0.897	1.58 (0.53, 4.72)	0.417	1.71 (0.50, 5.92)	0.394

8	26	11	42.31	21	8	38.10	17	6	35.29	0.84 (0.26, 2.72)	0.770	0.74 (0.21, 2.63)	0.646	0.89(0.23, 3.35)	0.859
9	21	9	42.86	16	7	43.75	12	6	50.00	1.04 (0.28, 3.85)	0.957	1.33 (0.32, 5.54)	0.692	1.29 (0.29, 5.77)	0.743
10	25	10	40.00	24	12	50.00	20	11	55.00	1.50 (0.48, 4.65)	0.483	1.83 (0.56, 6.03)	0.318	1.22 (0.37, 4.02)	0.741
11	19	7	36.84	10	5	50.00	17	8	47.06	1.71 (0.36, 8.08)	0.496	1.52 (0.40, 5.78)	0.536	0.89 (0.19, 4.24)	0.883
12	24	16	66.67	21	9	42.86	17	11	64.71	0.38 (0.11, 1.26)	0.112	0.92 (0.25, 3.39)	0.896	2.44 (0.65, 9.13)	0.184

* Odds ratios (ORs) compare 1-year survival between calendar years within the same month of diagnosis.

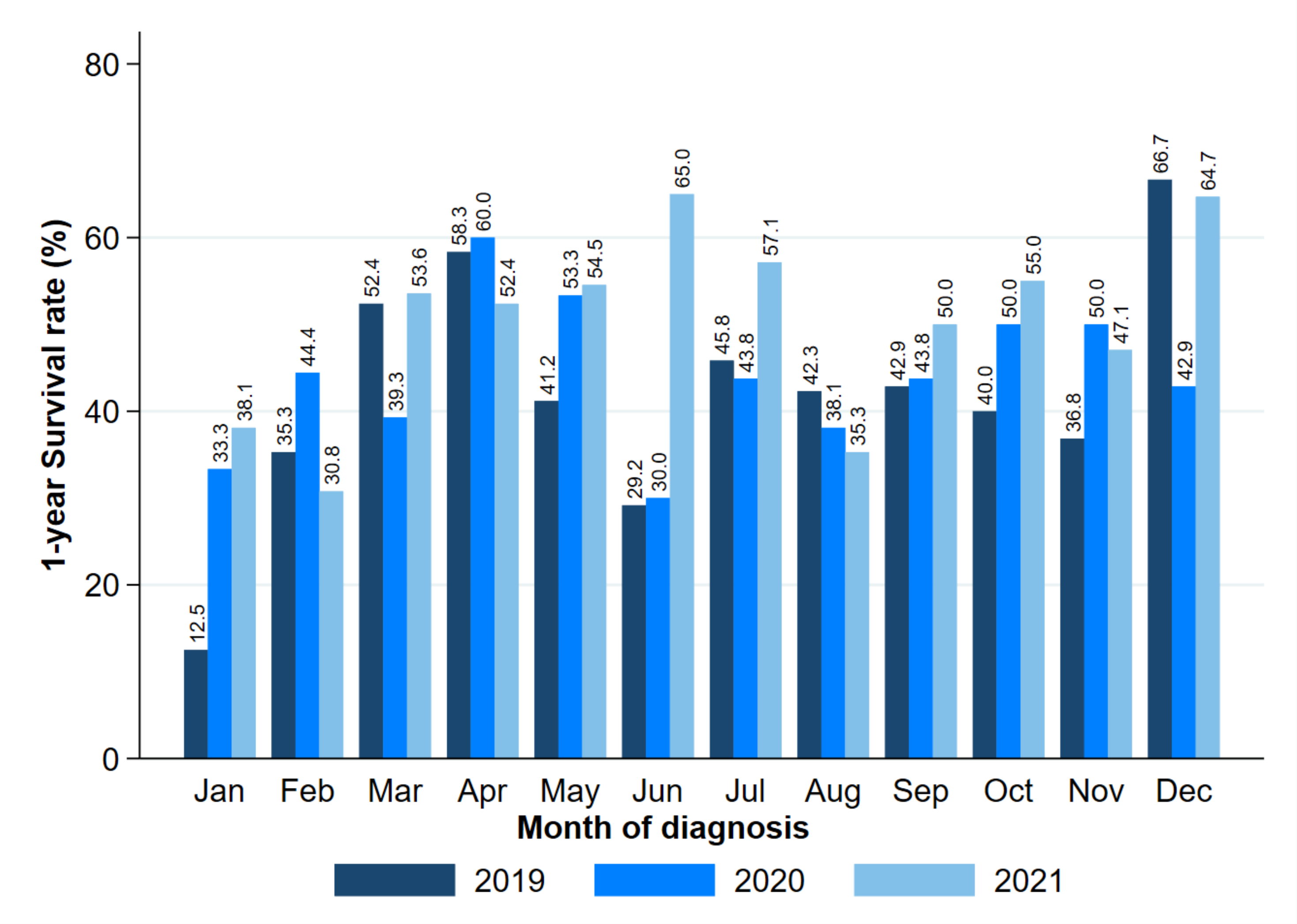

Figure S1. Monthly Variation in 1-Year Survival Rates by Year of Diagnosis (2019–2021)
